# Weather and mortality: a 10 year retrospective analysis of the Nouna Health and Demographic Surveillance System, Burkina Faso

**DOI:** 10.3402/gha.v5i0.19078

**Published:** 2012-11-23

**Authors:** Eric Diboulo, Ali Sié, Joacim Rocklöv, Louis Niamba, Maurice Yé, Cheik Bagagnan, Rainer Sauerborn

**Affiliations:** 1 Centre de Recherche en Santé de Nouna, Nouna, Burkina Faso; 2 Department of Epidemiology and Public Health, Swiss Tropical and Public Health Institute, Basel, Switzerland; 3 Department of Public Health and Clinical Medicine, Epidemiology and Global Health, Umea University, Umea, Sweden; 4 Institute of Public Health, Heidelberg University, Heidelberg, Germany

**Keywords:** weather, mortality, Burkina Faso, sub-Saharan Africa, Nouna HDSS, lag, time series, precipitation, temperature, climate change, vulnerability, susceptibility

## Abstract

**Background:**

A growing body of evidence points to the emission of greenhouse gases from human activity as a key factor in climate change. This in turn affects human health and wellbeing through consequential changes in weather extremes. At present, little is known about the effects of weather on the health of sub-Saharan African populations, as well as the related anticipated effects of climate change partly due to scarcity of good quality data. We aimed to study the association between weather patterns and daily mortality in the Nouna Health and Demographic Surveillance System (HDSS) area during 1999–2009.

**Methods:**

Meteorological data were obtained from a nearby weather station in the Nouna HDSS area and linked to mortality data on a daily basis. Time series Poisson regression models were established to estimate the association between the lags of weather and daily population-level mortality, adjusting for time trends. The analyses were stratified by age and sex to study differential population susceptibility.

**Results:**

We found profound associations between higher temperature and daily mortality in the Nouna HDSS, Burkina Faso. The short-term direct heat effect was particularly strong on the under-five child mortality rate. We also found independent coherent effects and strong associations between rainfall events and daily mortality, particularly in elderly populations.

**Conclusion:**

Mortality patterns in the Nouna HDSS appear to be closely related to weather conditions. Further investigation on cause-specific mortality, as well as on vulnerability and susceptibility is required. Studies on local adaptation and mitigation measures to avoid health impacts from weather and climate change is also needed to reduce negative effects from weather and climate change on population health in rural areas of the sub-Saharan Africa.

Weather has been found to have a bearing on mortality in most parts of the world, manifested through infectious diseases as well as numerous deaths related to, for example, heat waves (1–4). Existing literature, although mainly focused on urban settings, suggests differential weather-related mortality and morbidity between rural and urban populations. It is believed that urban populations are more affected than rural populations, especially by oppressive heat (5).

Despite indications of adaptation/acclimatization in warm regions, it has been suggested that urban populations in tropical climates may also be vulnerable to high temperatures (2). The population vulnerability to heat-related mortality is often characterized and modified by the underlying prevalence of temperature-sensitive diseases, the level of socioeconomic development, and the age structure of population (6).

Some studies have reported short-term associations between rainfall and mortality. Rainfall is known to be associated with, in particular, gastrointestinal/diarrheal diseases, which show increasing rates following floods or elevated amounts of rainfall (7, 8). However, also tropical vector-borne diseases, such as the malaria mosquitoes biting rate and the related human incidence rates, are exacerbated shortly after a rainfall event (3).

At present, and from now on, climate change resulting in extreme weather conditions is expected to have a marked impact on weather-related mortality (9). However, at present the knowledge of the impact and how to avoid harmful effects related to weather and extreme climatic events are sparse, particularly, in rural Africa. This article studies the association between weather and daily mortality in the Nouna Health and Demographic Surveillance System (HDSS) area in Burkina Faso.

## Objectives

The objectives of this study are:

1. To investigate the association between temperature, rainfall, and mortality in the Nouna HDSS.
2. To study the lag between weather variables and mortality.
3. To contrast the associations in groups of age and sex.

## Methods

### Study site

The HDSS site of the Centre de recherche en santé de Nouna (CRSN, Nouna Health Research Centre) is located in the Nouna health district's catchment area in northwest Burkina Faso, 300 km from the capital, Ouagadougou.

The current geographic extent of the HDSS comprises one district hospital and 14 peripheral health facilities.

**Fig. 1:**
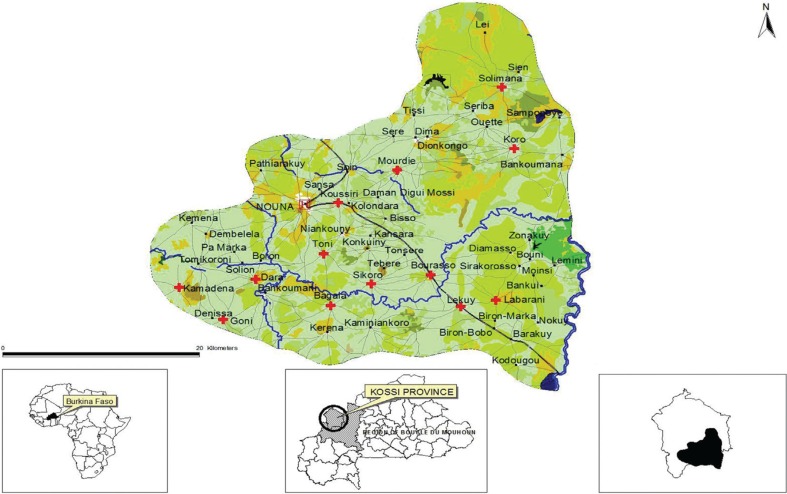
Map of Nouna Health and Demographic Surveillance System (HDSS's) catchment area.

The Nouna area is a dry orchard savannah with a sub-Saharan climate and a mean annual rainfall of 796 mm (range 483–1,083 mm) over the past five decades. The population size is about 90,000, settled over 1,775 km^2^. The population is rural and semi urban (essentially living in Nouna town) and almost exclusively subsistence farmers of the Marka, Bwaba, Samo, Mossi, and Foulani ethnic groups (Fig. 1).

The Nouna HDSS of CRSN has conducted regular population censuses since 1992 (baseline of individuals), maintained a vital-events-registration system, and performed routine verbal autopsy (VA) interviews (10).

The Nouna HDSS is a set of field and computing operations that handle the longitudinal follow-up of well-defined entities or primary subjects (individuals, households, and residential units) plus all related demographic and health outcomes within a clearly circumscribed geographical area.

The HDSS follow the entire population of a defined geographical area and monitors demographic and health characteristics over time. Initially, a census is carried out to define and register the target population where registered subjects are consistently and uniquely identified. Regular subsequent rounds of data collection at prescribed intervals (every 4 months) make it possible to register all new individuals, households, residential units and to update key attributes of existing subjects.

The core system monitors population dynamics through routine collection and processing of information on vital events such as births, deaths, and migrations which are the only demographic events that lead to any change in the initial size of the resident population.

In addition, the HDSS collects information on health outcomes (such as causes of death using VA, incidence, and prevalence of particular diseases of public health importance), performs routine surveillance of malaria indicators in randomly selected households, and conducts education and socio-economic surveys.

We observed clustering of deaths with unknown death date on the 1st and the 15th of each month. However, because we aim to study the weather-related mortality on a daily basis, we removed these dates from the analysis by imputing a missing value on these days. This made the total number of deaths at hand to study to decrease by 32% as can be seen in Table 1. Table 1 also shows the aggregated number of deaths over the study period in groups of age.

**Table 1:** Aggregated number of deaths over the study period (1999–2009) by age groups (after removing clustering of deaths on the 1st and 15th of each month)

Months	U5 (0–4)	All cause mortality Teenager Teenager (5–19)	Adults (20–59)	Elderly (60+)	Total
January	246	34	114	206	600
February	229	50	134	181	594
March	229	57	137	190	615
April	242	63	125	227	658
May	200	40	121	160	521
June	180	54	116	152	502
July	239	39	89	128	496
August	397	53	127	118	695
September	365	47	110	130	652
October	391	54	118	148	711
November	361	52	105	147	666
December	300	65	134	193	692

### Weather data

Data were collected from 10 onsite meteorological stations run by the Nouna Health Centre as well as from the nearest monitoring station associated with the World Meteorological Organization (WMO). Collection was done from 1999 to 2009 on a daily basis from the WMO station. The observations from the site-specific stations were compared to the one from the WMO station during the shorter period when the onsite stations were in use (2004–2009). Daily weather was aggregated from hourly measurements to daily mean, max and minimum temperature, as well as daily cumulative rainfall. Missing observations were not imputed. Lagged effects of daily weather were studied using lag strata of average meteorology respectively for lag 0–1, lag 2–6, and lag 7–13 to avoid problems arising from using highly correlated lags of weather variables in the same model.

Daily mean, maximum, and minimum temperature, as well as daily cumulative precipitation is presented in Table 2.

**Table 2:** Summary of weather data over the study period (1999–2009)

Months	Mean temperature (°C)	Minimum temperature (°C)	Maximum temperature (°C)	Mean precipitation (mm)
January	26.1	20.0	30.8	0
February	29.0	22.5	33.9	0
March	32.3	25.9	37.2	14.7
April	33.4	28.1	38.0	50.7
May	32.3	27.7	36.6	50.5
June	29.8	25.6	33.6	135
July	27.3	23.8	30.8	215.5
August	26.23	23.1	29.5	339.7
September	26.83	23.0	30.8	194.6
October	29.1	23.9	34.3	47.9
November	29.0	22.3	34.8	16.5
December	26.9	20.0	32.7	0

### Statistical analysis

We used a time series approach to study the association of weather variables with daily mortality series (11). Daily mortality was assumed to follow an over-dispersed Poisson distribution. Time trends were estimated with natural cubic spline function, allowing a degree of freedom (df) of five per year of data using the mgcv package in R, but without penalizing the complexity of the smooth function of time trends. The adjustment for time trends and seasonality allowed us to study how well weather variables predicted deviations in mortality from what is expected at a given time (season, year), that is, the short-term relationship between a weather stressor and succeeding mortality. In this way, the adjustment for time trends also adjusts for slowly varying changes in population size on a seasonal or annual basis.

Penalized smooth functions were used when estimating the exposure–response associations between lags of weather and daily mortality. This allowed the model to iteratively estimate the complexity of this relationship and enhance the fitting of a smoother relationship rather than noisy. These functions were allowed a maximum df of 10 before penalization. Linear exposure–response relationships were also estimated.

Because there was a large proportion of missing recordings of precipitation (see Table 2), we modelled the effects of the different weather stressors separately.

Models were evaluated on the basis of generalized cross validation (GCV) score. The GCV score is a rapidly computed metric that is based on a leave-one-observation-out method of maximizing the fit of the model through minimizing residual error. A smaller GCV corresponds to a better fit of the weather variables to the daily mortality data.

Sensitivity analyses of estimates were performed by changing the df per year of data from 5 to 8 in the spline function, estimating season and time trends, so as to assess the robustness of the estimates presented to the adjustment for time trends.

The fitted regression model was of the form:

$${\text{mortality}}_{t}\sim \text{Poisson}({\text{mean}}_{t})$$

$$\begin{array}{cc} {\text{log(mean}}_{t}\text{)=} & s\text{(weather lag 0}–1,\text{ df < 10)}\\ & +s\text{(weather lag 2}–6,\text{ df < 10)}\\ & +s\text{(weather lag 7}–13,\text{ df < 10)}\\ & +s\text{(time, df = 5 per year of data)} \end{array}$$

and

$$\begin{array}{cc} {\text{log(mean}}_{\text{t}}\text{) =} & \text{weather lag 0}–1+\text{weather lag 2}–6\\ & +\text{weather lag 7}–13,\\ & +s(\text{time},\text{df}=\text{5 per year of data}) \end{array}$$

where *t* denotes the time in days, *s* denotes a smooth cubic spline function, and df denotes the degrees of freedom.

## Results

The annual seasonal mortality patterns are described in Table 1. Overall, the monthly number of deaths was 61.68 over the study period. Weather was hottest in April with a minimum and maximum temperature of 28.1°C and 38.0°C, respectively, and coldest in January, with a minimum and maximum temperature of 20.0°C and 30.8° C (Table 2). The rainiest month was August with a mean precipitation of 339.7 mm (Table 2).

**Fig. 2:**
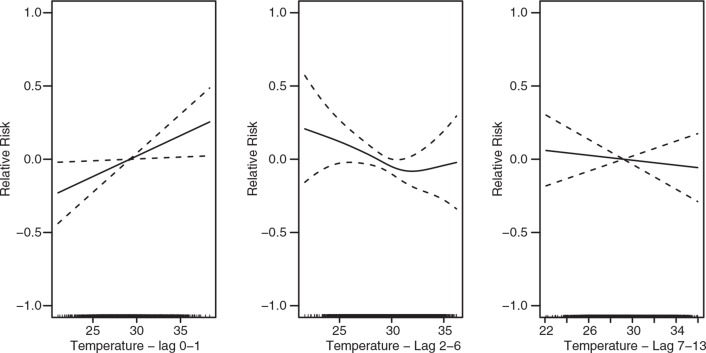
The association between temperature and all-cause and all age daily mortality in Nouna, Burkina Faso, over the lag 0–1, 2–6, and 7–13 (from left to right). The scale of the vertical axis is the log (relative risk [RR]), 95% confidence limits are shown as dotted lines.

**Fig. 3:**
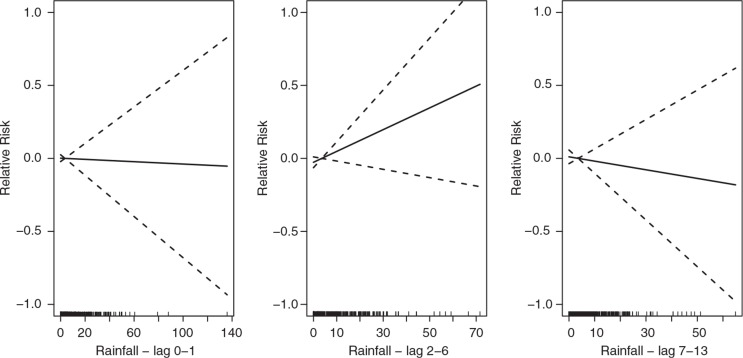
The association between precipitation and all-cause and all-age daily mortality in Nouna, Burkina Faso, over the lag 0–1, 2–6, and 7–13 (from left to right). The scale of the vertical axis is the log (relative risk [RR]), 95% confidence limits are shown as dotted lines.

For total all-age mortality, we estimated an approximate linear significant increase with increasing temperature in lag 0–1, and a slightly decreasing mortality (but not significantly) in lag 2–6, and lag 7–13 (Fig. 2). The increase in mortality in lag 0–1 corresponds to an approximate 50% increase in mortality over the range of temperature. In this group, rainfall is estimated as not being significantly related to mortality, but 2–6 days after rainfall mortality indicated an increase (Fig. 3). We note that the reliability of this test is lessened due to the large number of missing days with rainfall records.

The analysis using smooth curves show approximate linear relationships overall. As linear estimates the group of all-ages appear to experience significant elevated risks to temperature increases in lag 0–1 only. This association is particularly apparent in the age group of 0–4 (Table 3). Rainfall shows no significant association with mortality in this group, however.

**Table 3:** Relative risk (RR) in % associated with a 1°C increase of temperature per lag strata

	Lag 0–1		Lag 2–6		Lag 7–13	
			
Age group	RR	CI (95%)	RR	CI (95%)	RR	CI (95%)
0–4	**3.7**	(0.3, 7.3)	−1.6	(−6.0, 3.0)	0.4	(−4.2, 5.2)
5–19	3.2	(−1.9, 8.6)	1.6	(−5.2, 8.9)	−0.4	(−7.1, 6.9)
20–59	2.3	(−1.6, 6.5)	−4.2	(−9.1, 0.9)	0.3	(−4.9, 5.7)
60+	1.1	(−2.4, 4.6)	−2.6	(−7.1, 1.7)	−4	(−8.3, 0.5)
All ages	**2.6**	(0.1, 5.2)	−2.4	(−5.5, 0.9)	−1	(−4.3, 2.3)
Men	2.5	(−0.5, 5.6)	−2.9	(−6.7, 0.1)	1.3	(−2.7, 5.5)
Women	2.8	(−0.5, 6.1)	−1.8	(−5.8, 2.4)	−3.6	(−7.6, 0.6)

Estimates significant at the 95% level are marked as bold.

Tables 3 and 4 also describe the relationship estimated between temperature and rainfall and daily deaths in the age group of 20–59 years. In this age group, increasing temperature indicates no association with mortality over the lags studied, however, rainfall shows increasing mortality but with decreasing levels in the lag 7–13.

**Table 4:** Relative risk (RR) in % associated with a 1 mm increase of rainfall per lag strata

	Lag 0–1		Lag 2–6		Lag 7–13	
			
Age group	RR	CI (95%)	RR	CI (95%)	RR	CI (95%)
0–4	0.01	(−0.8, 0.8)	0.06	(−1.2, 1.4)	0.2	(−1.4, 1.8)
5–19	0.02	(−1.3, 1.3)	**2.4**	(0.5, 4.5)	0.6	(−2.0, 0.6)
20–59	−0.23	(−1.5, 1.1)	0.3	(−1.7, 2.3)	**−3.3**	(−2.1, −0.7)
60+	−0.05	(−1.08, 0.1)	**1.9**	(0.3, 1.9)	0.01	(−2.1, 2.2)
All ages	−0.04	(−0.7, 0.6)	0.8	(−0.3, 1.8)	−0.3	(−1.6, 1.0)
Men	−0.07	(−0.1, 0.8)	0.8	(−0.6, 2.1)	−0.5	(−2.1, 1.2)
Women	−0.01	(−0.8, 0.8)	0.8	(−0.5, 2.0)	−0.1	(−1.7, 1.5)

Estimates significant at the 95% level are marked as bold.

The group of 5–19 years of age appears more sensitive to high rainfall. Table 4 describes an increasing mortality with increasing levels of rainfall in the lag period of 7–13 days in this age group.

**Fig. 4:**
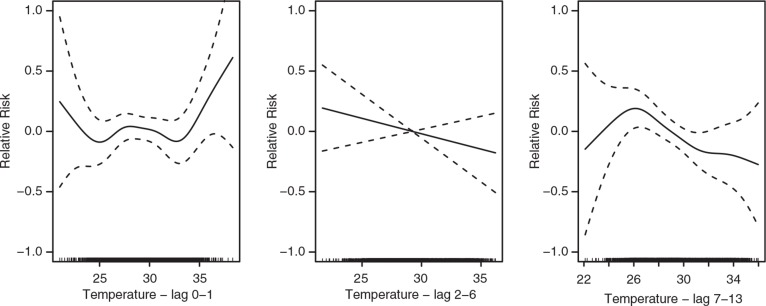
The association between temperature and elderly (60+ of age) daily mortality in Nouna, Burkina Faso, over the lag 0–1, 2–6, and 7–13 (from left to right). The scale of the vertical axis is the log (relative risk [RR]), 95% confidence limits are shown as dotted lines.

The elderly population (60+ of age) in Nouna appears sensitive to both extreme high and low temperatures in lag 0–1 (Fig. 4). However, when studied linearly no significant associations are estimated. More intensive rainfall in the lag 2–6 days is significantly associated with mortality showing a substantial increase in mortality following such events (Table 4).

Sensitivity analyses showed no changes in the relationship estimated between temperature and daily deaths (Table 5). However, the estimated relationship between rainfall and daily deaths indicates no further association with mortality in the group of 5-15 years of age and elderly population (Table 6).

**Table 5:** Sensitivity analysis for df=8: Relative risk (RR) in % associated with a 1°C increase of temperature per lag strata

	Lag 0–1		Lag 2–6		Lag 7–13	
			
Age group	RR	CI (95%)	RR	CI (95%)	RR	CI (95%)
0–4	**4.04**	(4.0, 7.8)	−2.32	(−7.0, 2.6)	−1.3	(−6.6, 4.3)
5–19	1.23	(−4.0, 6.8)	−3.54	(−10.4, 3.9)	−7.2	(−14.7, 0.9)
20–59	2.6	(−1.6, 6.9)	−4.14	(−9.4, 1.4)	2.4	(−3.9, 9.2)
60+	1.8	(−1.8, 5.6)	−0.8	(−5.5, 4.1)	−1.7	(−6.8, 3.7)
All ages	**2.9**	(0.29, 5.6)	−2.3	(−5.7, 1.2)	−1.0	(−4.9, 2.9)
Men	2.89	(−0.3, 6.2)	−2.7	(−6.7, 1.6)	1.3	(−3.4, 16.3)
Women	2.89	(−0.5, 6.4)	−2.0	(−6.3, 2.5)	−3.7	(−8.4, 1.3)

Estimates significant at the 95% level are marked as bold.

**Table 6:** Sensitivity analysis for df=8: Relative risk (RR) in % associated with a 1 mm increase of rainfall per lag strata

	Lag 0–1		Lag 2–6		Lag 7–13	
			
Age group	RR	CI (95%)	RR	CI (95%)	RR	CI (95%)
0–4	0.06	(−0.8, 0.9)	0.2	(−1.4, 1.8)	0.4	(−1.6, 2.3)
5–19	−0.7	(−2.2, 0.8)	0.7	(−2.1, 3.7)	−1.2	(−4.3, 2.0)
20–59	−0.1	(−1.5, 1.2)	0.6	(−1.8, 3.0)	−3.2	(6.1, −0.2)
60+	−0.1	(−1.3, 1.0)	1.8	(−0.2, 3.9)	0.02	(−2.5, 2.7)
All ages	−0.8	(−0.8, 0.6)	0.7	(−0.6, 2.0)	−0.3	(−1.9, 1.2)
Men	2.9	(−0.3, 6.2)	−2.7	(−6.7, 1.6)	1.3	(−3.4, 6.3)
Women	0.05	(−0.8, 0.9)	1.1	(−0.5, 2.6)	0.4	(−1.5, 2.4)

## Discussion

We found profound associations between higher temperature and daily mortality in the Nouna HDSS, Burkina Faso. The short-term direct heat effects lag 0–1 was particularly strong among the younger population, but also apparent in all ages. We also found coherent strong associations between rainfall events and daily mortality delayed 2–6 days, particularly, in the elderly populations. Also, interestingly, temperature indicated a U-shaped relationship with mortality over lag 0–1 in the elderly population (60+ of age). This resembles the relationship between elderly populations and mortality in developed countries today (1, 12).

Future studies should investigate these associations in cause-specific groups to better understand the under-lying chain of events that are potentially involved in causing harmful effects from weather among the population of Nouna HDSS.

The increasing mortality seen in lag 2–6 with increasing rainfall could be related to pathogen contamination of fresh water, and intensified biting rate of mosquito and transmission of malaria (13). The increasing mortality with increasing temperature in lag 0–1 is most likely a heat effect known to exacerbate a wide range of communicable and non-communicable diseases (14). The slight decrease in mortality in lag 2–6 and lag 7–13 may be related to effects from cold exposure known to correlate to cardiovascular and respiratory diseases (15). In general, temperature effects are known to be exacerbated and increase with age through deterioration of the body's thermoregulation system and the ability to sense and act on heat and cold impulses (16).

Future studies should investigate who is vulnerable and susceptible to the effects from weather in more detail in order to target populations and individuals at more increased risk and relative risk (RR) when developing adaptive measures to protect against harmful effects from weather and climate changes.

At present, childhood mortality seems to be most affected by high temperature and children suffer the most from extreme heat conditions resulting from climate change. If this continues, it may partly hinder the overall aim of reducing child mortality.

These results indicate that rural populations in sub-Saharan Africa are likely to experience harmful effects from increasing heat levels from climate change as suggested by the IPCC (17).

However, there are several limitations in this study. First, we used population-level exposures to temperature and rainfall, which within the HDSS can vary. In particular, rainfall is often more heterogeneous in space. However, due to the lack of spatial and temporal finely resolved data such exposure differences cannot be taken into account. Moreover, there was a large number of missing observations of, primarily, rainfall but also temperature. This will have reduced the statistical reliability of the study, but is unlikely to have caused any systematic errors. Furthermore, the clustering of events on the 1st and 15th of each month also weakened the study, but there is currently no reason to suspect that the events that were removed caused any systematic bias in the estimates.

## Conclusion

Our study highlighted that the population in Nouna, Burkina Faso, experience short-term increases in mortality in relation to specific meteorological events. In particular, those under five years of age appear more susceptible to hot temperatures, while the elderly population is more susceptible to increasing levels of rainfall. However, the elderly population appeared to be affected by both low and high temperatures – resembling a u-shaped relationship similar to those estimated on the elderly populations in developed cities. However, future studies are needed to confirm this.

Overall, mortality patterns in the Nouna HDSS appear to be closely related with short-term weather conditions; hence, further investigation on cause-specific mortality, vulnerability, and susceptibility factors to better understand the particular effects of weather and climate change on population health in rural areas of sub-Saharan Africa is required.
